# The Association Between Triglyceride-Glucose Index as a Marker of Insulin Resistance and the Risk of Breast Cancer

**DOI:** 10.3389/fendo.2021.745236

**Published:** 2021-10-11

**Authors:** Sonar Soni Panigoro, Noorwati Sutandyo, Fiastuti Witjaksono, Nurjati Chairani Siregar, Ramadhan Ramli, Ririn Hariani, Eko Adhi Pangarsa, Yan Wisnu Prajoko, Niken Puruhita, William Hamdani, Dimas Bayu, Mardiana Madjid, Dedy Yulidar, Jane Estherina Fransiska, Retno Widyawati, Effif Syofra Tripriadi, Wiwit Ade F. W., Dewi Krisna Yunda, Raymond Pranata

**Affiliations:** ^1^Department of Surgical Oncology, Dr. Cipto Mangunkusumo Hospital, Faculty of Medicine, Universitas Indonesia, Jakarta, Indonesia; ^2^Department of Hematology and Medical Oncology, Dharmais Hospital National Cancer Center, Jakarta, Indonesia; ^3^Department of Nutrition, Dr. Cipto Mangunkusumo Hospital, Faculty of Medicine, Universitas Indonesia, Jakarta, Indonesia; ^4^Department of Pathological Anatomy, Dr. Cipto Mangunkusumo Hospital, Faculty of Medicine, Universitas Indonesia, Jakarta, Indonesia; ^5^Department of Surgical Oncology, Dharmais Hospital National Cancer Center, Jakarta, Indonesia; ^6^Department of Nutrition, Dharmais Hospital National Cancer Center, Jakarta, Indonesia; ^7^Department of Hematology and Medical Oncology, Dr. Kariadi General Hospital, Faculty of Medicine, Diponegoro University, Semarang, Indonesia; ^8^Department of Surgical Oncology, Dr. Kariadi General Hospital, Faculty of Medicine, Diponegoro University, Semarang, Indonesia; ^9^Department of Nutrition, Dr. Kariadi General Hospital, Faculty of Medicine, Diponegoro University, Semarang, Indonesia; ^10^Department of Surgical Oncology, Wahidin Sudirohusodo General Hospital, Faculty of Medicine, Hasanuddin University, Makassar, Indonesia; ^11^Division of Hematology-Medical Oncology, Department of Internal Medicine, Hasanuddin University General Hospital, Faculty of Medicine, Hasanuddin University, Makassar, Indonesia; ^12^Department of Nutrition, Faculty of Medicine, Wahidin Sudirohusodo General Hospital, Hasanuddin University, Makassar, Indonesia; ^13^Department of Surgical Oncology, Prof. Dr. WZ Johannes General Hospital, Kupang, Indonesia; ^14^Division of Hematology and Medical Oncology, Department of Internal Medicine, Prof. Dr. WZ Johannes General Hospital, Kupang, Indonesia; ^15^Department of Pathological Anatomy, Prof. Dr. WZ Johannes General Hospital, Kupang, Indonesia; ^16^Department of Surgical Oncology, Arifin Achmad General Hospital, Faculty of Medicine, Riau University, Pekanbaru, Indonesia; ^17^Department of Pathological Anatomy, Arifin Achmad General Hospital, Faculty of Medicine, Riau University, Pekanbaru, Indonesia; ^18^Department of Nutrition, Arifin Achmad General Hospital, Faculty of Medicine, Riau University, Pekanbaru, Indonesia; ^19^Department of Nutrition, Nutrition Cancer Reaserch Team, Dharmais Hospital National Cancer Center, Jakarta, Indonesia

**Keywords:** breast cancer, insulin resistance, triglyceride, glucose, insulin

## Abstract

**Background:**

This study aims to evaluate the association and dose-response between triglyceride-glucose (TyG) index and breast cancer.

**Method:**

This is a multicenter case-control study conducted in six public referral hospitals in Indonesia. Cases are individuals aged 19 years or above who were diagnosed with breast cancer within 1 year of diagnosis, based on histopathology and immunohistochemistry. Controls were recruited from corresponding hospitals. TyG index was determined by the formula: ln (fasting TG [mg/dl] × fasting glucose [mg/dl]).

**Results:**

There were 212 participants in the breast cancer group and 212 participants in the control group. TyG index was higher in patients with breast cancer (median 8.65 [7.38, 10.9] vs. 8.30 [7.09, 10.84], *p* < 0.001). When compared with TyG quartile of Q1, Q4 was associated with an OR of 2.42 (1.77, 3.31), *p* < 0.001, Q3 was associated with an OR of 1.53 (1.21, 1.93), *p* < 0.001, Q2 was associated with an OR of 1.39 (1.12, 1.73), *p* = 0.002 for the risk of breast cancer. The dose-response relationship was nonlinear (*p* < 0.001). On univariate analysis, smoking (OR 2.15 [1.44, 3.22], *p* < 0.001), use of contraception (1.73 [1.15, 2.60], *p* = 0.008), alcohol consumption (OR 2.04 [0.96, 4.35], *p* = 0.064), and TyG Index >8.87 (OR 3.08 [1.93, 4.93], *p* < 0.001) were associated with risk of breast cancer. Independently associated with increased risk of breast cancer included smoking (OR 1.93 [1.23, 3.01], *p* = 0.004), use of contraception (OR 1.59 [1.02, 2.48], *p* = 0.039), and TyG Index >8.87 (OR 2.93 [1.72, 4.98], *p* < 0.001)

**Conclusion:**

TyG index was associated with breast cancer in a nonlinear dose-response fashion.

## Introduction

Breast cancer is the most common cancer worldwide (1, 2). There were an estimated 2.3 million new cases of female breast cancer out of 19.3 million new cases of cancer (i.e., breast cancer represents 11.7% of all new cancer cases) worldwide in 2020 (2). Breast cancer accounts for one in six cancer deaths and has become the leading cause of cancer death in the majority of countries. This trend also occurs in Indonesia where the incidence rate of breast cancer is 44.0 and mortality rate is 15.3 out of 100,000 (2, 3). Breast cancer is associated with several risk factors which numerous studies have investigated (4–7). Risk factors are commonly differentiated into two categories namely nonmodifiable and modifiable risk factors. The nonmodifiable risk factors consist of age, age of menarche, genetic factors, family history, and history of breast cancer, while the modifiable category includes weight status, fat intake, parity, breastfeeding status, alcohol consumption, smoking habit, and the use of contraception (4–6, 8).

Recent studies suggest that insulin resistance was associated with breast cancer and may affect its prognosis (9, 10). Euglycemic-hyperinsulinemic clamp (clamp-IR) is the gold standard for IR diagnosis (11); however, its use is impractical (12). Triglyceride-glucose (TyG) index is a reliable surrogate marker of insulin resistance (13), which is calculated by formula comprising fasting glyceride and glucose, which is usually assessed in apparently healthy individuals (14). Thus, risk stratification for breast cancer using TyG index is practical, feasible, and cost-effective. Although ideally evaluated using a prospective cohort study, this case-control study may provide early evidence regarding the association between insulin resistance and breast cancer. This study aims to evaluate the association between TyG index and breast cancer and assess the dose-response between TyG index and breast cancer.

## Patients and Methods

### Study Population

This was a multicenter case-control study conducted in six public referral hospitals in Indonesia, namely, Ciptomangunkusumo Hospital in Jakarta, Dharmais Cancer Center in Jakarta, Arifin Achmad General Hospital in Pekanbaru, Dr. Kariadi Hospital in Semarang, Wahidin Sudirohusodo Hospital in Makassar, and Prof. Dr. WZ. Johannes Hospital in Kupang. Recruitment of study participants was performed consecutively between April to August 2019. A total of 432 women consisting of 216 cases and 216 controls were recruited. The sample size was derived from proportion estimate of two population with the formula (Z1−α/2√2PQ + Z1−β√P1Q 1+P2Q2)^2^/(P1−P2)^2^.

The inclusion criteria for the case group are as follows (1): individuals aged 19 years or above who were diagnosed with breast cancer based on histopathology and immunohistochemistry between April to August 2019. The maximum year postdiagnosis was 1 year (2), patients with breast cancer that has not received therapy, (3) can read, understand, and provide consent, and (4) complete medical record and paraffin block. Those with incomplete questionnaire data were excluded. Controls were recruited from corresponding hospitals. Inclusion criteria for the control group were as follows: (1) women aged 19 years or above (matched 5 years), (2) in healthy conditions based on anamnesis and physical examination results, (3) no evidence of cancer or history of cancer, and (4) no evidence of chronic disease. Most individuals recruited as controls were hospital employees. The study was approved by Ethical Committee of Health Research in the Faculty of Medicine, Universitas Indonesia, Rumah Sakit Cipto Mangunkusumo, Jakarta, Indonesia (450/UN2.F1/ETIK/2018).

### Measurements

Data were collected through medical records and a self-administered structured questionnaire. The questionnaire included information on age at menarche, smoking history, reproductive risk factors (i.e., breastfeeding and use of contraception), family history of malignancy, alcohol consumption, and nutrition intake.

TyG index was determined by the formula: ln (fasting TG [mg/dl] × fasting glucose [mg/dl]) (14).

Weight and height were measured to the nearest 0.1 kg and 0.5 cm according to standardized procedures. The study participants wore light clothes and no shoes during the measurement. Body mass index (BMI) was calculated as weight in kilograms divided by the square of height in meters. BMI was classified according to WHO into underweight (<18.5 kg/m^2^), normal weight (18.5–24.9 kg/m^2^), and overweight and obese (>24.9 kg/m^2^).

Menarche was defined as the age when the first menstruation occurred. The use of contraception, breastfeeding history, smoking status, and alcohol consumption were defined from the questionnaire. Participants were classified as using contraception/breastfeeding/having smoking history/consuming alcohol if they answered “yes” to the question “have you ever used contraception/breastfed/smoked cigarettes/drunk alcohol during your entire life?”. Those who answered yes were then asked for the duration of using contraception/breastfeeding/having smoking history/consuming alcohol. Type of contraception was also asked to those who ever used contraception.

### Statistical Analysis

Categorical data are presented as proportions. The distribution of continuous data was inspected using QQ plots, histograms, Kolmogorov-Smirnoff, and Shapiro-Wilk test. Normally distributed data are presented as means and standard deviations (SD), while nonparametric data are presented as median, minimum, and maximum values (median (min-max)). Comparison of categorical variables was tested using Chi-square test. Continuous variables were compared using independent *t*-test or Mann-Whitney *U* test, where appropriate.

The TyG was divided into four quartiles, and the odds ratios (ORs) for each quartile were calculated using the first quartile as the reference. Restricted cubic spline model was constructed using four knots at 7.9, 8.3, 8.7, and 9.4; nonlinearity of the dose-response curve was also assessed. The ORs for TyG index and breast cancer were calculated using logistic regression. A cutoff point for TyG index was set at the beginning of the fourth quartile for multivariate analysis. Multivariate logistic regression was used to test the association between breast cancer and each independent variable. The results are presented as ORs with 95% confidence intervals (CIs). Statistical significance was defined as *p* < 0.05. Data were managed and analyzed using SPSS 25.0 (IBM, Armonk, US) and STATA^®^ version 16 (StataCorp, College Station, TX, USA).

## Results

### Baseline Characteristics

There were 212 participants in the breast cancer group and 212 participants in the control group. The baseline characteristics of the participants in this study can be seen in **Table 1**. The distribution of BMI categories was more or less the same, except in the underweight group, in which there was a significantly higher breast cancer patient in the underweight group.

**Table 1:** Baseline characteristics of study participants.

	Breast cancer (+)	Breast cancer (−)	*p*-Value
	*n* = 212	*n* = 212	
Age (year)	48 (22–78)	46 (22–75)	0.001
Smoking	100 (49.3)	65 (31.1)	0.001
Age at menarche (year)	13 (9–19)	13 (8–18)	<0.001
Breastfeeding ≥12 months	81 (42)	87 (43.7)	0.726
Use of contraception	96 (48.7)	67 (35.4)	0.008
Family history of malignancy	35 (16.6)	45 (21.5)	0.197
Alcohol consumption	21 (10.1)	11 (5.2)	0.060
Body mass index (kg/m^2^)			
Underweight	15 (7.2)	2 (1)	0.001
Normal	60 (28.8)	47 (22.5)	0.137
Overweight	39 (18.8)	46 (22)	0.409
Obese	94 (45.2)	114 (54.5)	0.056
TyG index	8.65 (7.38–10.9)	8.30 (7.09–10.84)	<0.001
Q1 (7.09–8.12)	28 (13.2)	73 (34.4)	<0.001
Q2 (8.13–8.47)	52 (24.5)	56 (26.4)	0.656
Q3 (8.48–8.86)	57 (26.9)	51 (24.1)	0.504
Q4 (8.87–10.90)	75 (35.4)	32 (15.1)	<0.001
Total cholesterol	201.5 (71–343)	206 (113–561)	0.190
LDL	135 (39–268)	136 (67–268)	<0.001

TyG index, triglyceride-glucose index.

### Triglyceride-Glucose Index and Breast Cancer

**Figure 1:**
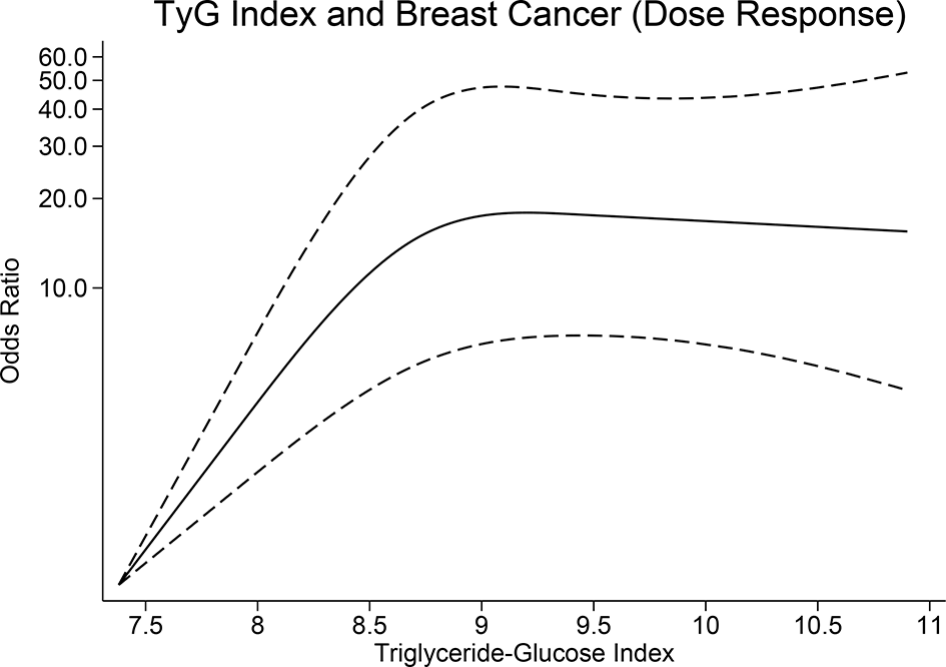
Dose-response relationship between triglyceride-glucose index and breast cancer.

TyG index was higher in patients with breast cancer (median 8.65 [7.38, 10.9] vs. 8.30 [7.09, 10.84], *p* < 0.001). The patients were divided into four quartiles based on the TyG index, namely, Q1 (7.09–8.12), Q2 (8.13–8.47), Q3 (8.48–8.86), and Q4 (8.87–10.90); comprising of 101, 108, 108, and 107 patients, respectively. When compared with TyG quartile of Q1, Q4 was associated with an OR of 2.42 (1.77, 3.31), *p* < 0.001, Q3 was associated with an OR of 1.53 (1.21, 1.93), *p* < 0.001, Q2 was associated with an OR of 1.39 (1.12, 1.73), *p* = 0.002 for the risk of breast cancer (**Table 2**). There was a non-linear relationship between TyG index and breast cancer (*p* < 0.001) (**Figure 1**).

**Table 2:** Quartiles of triglyceride-glucose index and the risk of breast cancer.

TyG index quartiles	Odds ratio	*p*-Value
Q1 (7.09–8.12) [*n* = 101]	Reference value	Reference value
Q2 (8.13–8.47) [*n* = 108]	1.39 (1.12, 1.73)	*p* = 0.002
Q3 (8.48–8.86) [*n* = 108]	1.53 (1.21, 1.93)	*p* < 0.001
Q4 (8.87–10.90) [*n* = 107]	2.42 (1.77, 3.31)	*p* < 0.001

TyG index, triglyceride-glucose index.

### Univariate Analysis

On univariate analysis, variables that contribute to increased risk of breast cancer were smoking (OR 2.15 [1.44, 3.22], *p* < 0.001), use of contraception (1.73 [1.15, 2.60], *p* = 0.008), alcohol consumption (OR 2.04 [0.96, 4.35], *p* = 0.064), and TyG Index >8.87 (OR 3.08 [1.93, 4.93], *p* < 0.001) (**Table 3**).

**Table 3:** Univariate and multivariate analysis of risk factors for breast cancer.

	Univariate analysis	Multivariate analysis
Age >60 years old	1.64 [0.85, 3.16], *p* = 0.142	1.46 [0.71, 3.04], *p* = 0.305
Early menarche (<12 years old)	1.19 [0.62, 2.27], *p* = 0.599	1.32 [0.64, 2.74], *p* = 0.450
Family history of malignancy	0.73 [0.44, 1.18], *p* = 0.197	0.66 [0.38, 1.15], *p* = 0.143
Smoking	2.15 [1.44, 3.22], *p* < 0.001	1.93 [1.23, 3.01], *p* = 0.004
Use of contraception	1.73 [1.15, 2.60], *p* = 0.008	1.59 [1.02, 2.48], *p* = 0.039
Alcohol consumption	2.04 [0.96, 4.35], *p* = 0.064	2.24 [0.97, 5.18], *p* = 0.059
TyG index >8.87	3.08 [1.93, 4.93], *p* < 0.001	2.93 [1.72, 4.98], *p* < 0.001
Total cholesterol > 200 mg/dl	0.86 [0.59, 1.26], *p* = 0.436	0.87 [0.53, 1.41], *p* = 0.565
LDL >100 mg/dl	0.68 [0.39, 1.18], *p* = 0.164	0.75 [0.38, 1.47], *p* = 0.400

TyG index, triglyceride-glucose index; LDL, low-density lipoprotein.

### Multivariate Analysis

On multivariate analysis, variables that were independently associated with increased risk of breast cancer included smoking (OR 1.93 [1.23, 3.01], *p* = 0.004), use of contraception (OR 1.59 [1.02, 2.48], *p* = 0.039), and TyG Index >8.87 (OR 2.93 [1.72, 4.98], *p* < 0.001) (**Table 3**).

## Discussion

This study indicates that TyG index was associated with breast cancer in a nonlinear dose-response fashion. TyG index >8.87 was independently associated with a threefold risk of breast cancer. Although there was no statistically significant difference in terms of overweight and obesity between the two groups, TyG index, which is a marker of insulin resistance, was higher in patients with breast cancer.

Hyperinsulinemia has been shown to be a risk factor for breast cancer as shown by previous studies using fasting insulin or c-peptide measurement (15–18). A Post Genome-Wide Gene–Environment Interaction Study identify insulin resistance single-nucleotide polymorphisms (SNPs) in combination with lifestyle as a synergistic factors for the risk of breast cancer (9). A study involving 22,837 postmenopausal women found that insulin resistance measured using homeostatic model assessment for insulin resistance in postmenopausal women were associated with higher incidence of breast cancer and mortality (10). Interestingly, a study by Kabat et al. indicates that although elevated serum insulin was associated with breast cancer, glucose alone was not (18). A study by Zhu et al. on 2,536 patients with breast cancer and 2528 patients with benign breast disease showed that insulin and insulin resistance was associated with breast cancer risk in Chinese women (19).

Gunter et al. showed that insulin resistance, and not adiposity per se, is a risk factor for postmenopausal breast cancer (20). Several reports indicate that overweight with normal insulin sensitivity does not have increased risk for cardiovascular disease (21–23), which might also be the case for breast cancer. In our study, alike to that of the study of Gunter et al. (20), TyG index was independently associated with breast cancer despite similar baseline BMI characteristics. Since the dose-response relationship between TyG index and risk of breast cancer was nonlinear, a TyG index >8.87 which marks the beginning of the fourth quartile was used as the cutoff point.

This present study showed that contraception use was positively associated with the risk of breast cancer, although statistical significance was lost in the multivariate analyzed. This finding was well-reported in previous studies (24–26). In the present study, we did not distinguish the type of hormonal contraception. Several studies indicated no significant differences regarding the type of oral contraception being used by individuals with breast cancer (24, 25), while another study in the USA shows that progestin-only pill consumption was not correlated with the risk of breast cancer (27). Moreover, some studies found that the duration of hormonal contraception use was correlated with increased risk of breast cancer (24, 25).

Although there is tendency towards increased risk of breast cancer related to alcohol consumption in the present study, it did not reach statistical significance. Previous studies have shown that alcohol was associated with breast cancer (28–32). A meta-analysis reported that there is a significant association between light drinking and breast cancer (32). Another study identified that increased alcohol intake in postmenopausal women was linked to a higher risk of breast cancer (31). It has been widely accepted that the biological mechanism underlying the correlation between alcohol and breast cancer is through the effects of alcohol on circulating estrogen levels and thus mostly associated with positive estrogen receptor breast cancer (28, 33). This study did not stratify the amount of alcohol consumption, the association might be dose related.

We did not found association of breast cancer with age at menarche and breastfeeding. Yet, the association between age at menarche and breast cancer has been established in previous studies. Several studies showed that early age at menarche was associated with increased risk of breast cancer (6, 34–36). A meta-analysis of 117 studies reported that every year younger at menarche led to increasing the risk of breast cancer by the odds of 1.050 (95% CI 1.044–1.057, *p* < 0.0001) (35). In the present study, the crude analysis indicated a significant association between age at menarche and breast cancer, while such association was not shown in the adjusted model. Lack of power may be a reason for this null association.

The association between breastfeeding and breast cancer has been contradictive. The present study found a null association of breastfeeding with breast cancer. This finding is in line with several studies (6, 37). In contrast, other studies reported that breastfeeding has a protective effect on breast cancer (8, 38, 39). The risk of breast cancer who breastfed exclusively was 28% lower compared with those who had never breastfed (38). One of the biological explanations for this association is that prolonged breastfeeding leads to decreased exposure to the cyclic reproductive hormones (39).

A few studies have shown association between smoking and breast cancer (40, 41). This study support the link between smoking and breast cancer. A study in the UK found that women who smoked have a higher risk of breast cancer, particularly those who smoked >5 cigarettes per day, 10+pack-years of use (40). This finding indicated that relationship between smoking and breast cancer is stronger in a dose-response pattern, rather than as a binary association.

### Limitations

One of the limitations was due to self-reported measurements used in the study, recall bias and social desirability bias might have occurred. Furthermore, selection bias might have occurred due to hospital-based study design. In addition, as different histological subtypes of breast cancer might have different risk factors, a larger longitudinal study is needed to assess factors associated with histological subtypes of breast cancer. Finally, levels of triglycerides and glucose are variable and are related to the time since the last meal.

## Conclusion

TyG index was associated with increased risk for breast cancer in a non-linear fashion. Further prospective studies are required to confirm this finding.

## Data Availability Statement

The raw data supporting the conclusions of this article will be made available by the authors, without undue reservation.

## Ethics Statement

The studies involving human participants were reviewed and approved by Ethical Committee of Health Research in the Faculty of Medicine, Universitas Indonesia, Rumah Sakit Cipto Mangunkusumo, Jakarta, Indonesia (450/UN2.F1/ETIK/2018). The patients/participants provided their written informed consent to participate in this study.

## Author Contributions

SP: conceptualization, design, data curation, investigation, and writing (original draft). NS: data curation, investigation, and writing (original draft). FW: data curation, investigation, and writing (original draft). NS: data curation, investigation, and writing (original draft). RR: data curation, investigation, and writing (review and editing). RH: data curation, investigation, and writing (review and editing). EP: data curation, investigation, and writing (review and editing). YP: data curation, investigation, and writing (review and editing). NP: Data curation, investigation, and writing (review and editing). WH: data curation, investigation, and writing (review and editing). DB: data curation, investigation, and writing (review and editing). MM: data curation, investigation, and writing (review and editing). DY: data curation, investigation, and writing (review and editing). JF: data curation, investigation, writing (review and editing). RW: data curation, investigation, and writing (review and editing). ET: data curation, investigation, and writing (review and editing). WF: data curation, investigation, and writing (review and editing). DY: data curation, investigation, and writing (review and editing). RP: conceptualization, investigation, formal analysis, and writing (original draft). All authors contributed to the article and approved the submitted version.

## Conflict of Interest

The authors declare that the research was conducted in the absence of any commercial or financial relationships that could be construed as a potential conflict of interest.

## Publisher’s Note

All claims expressed in this article are solely those of the authors and do not necessarily represent those of their affiliated organizations, or those of the publisher, the editors and the reviewers. Any product that may be evaluated in this article, or claim that may be made by its manufacturer, is not guaranteed or endorsed by the publisher.
